# *Elaeocarpus sylvestris* (Lour.) Poir.: Phytochemistry and Pharmacological Potential—A Review

**DOI:** 10.3390/molecules31081299

**Published:** 2026-04-16

**Authors:** Sultan Mehtap Büyüker, Khizar Abdullah Khan, Abdul Qadeer Khan Khalil, Imran Khan, Shah Jahan, Muhammad Adil, Khalid M. Al-Rohily, Abdulmoneem H. Al-Khamees, Atif Ali Khan Khalil

**Affiliations:** 1 Department of Pharmaceutical Toxicology, School of Pharmacy, Istanbul Medipol University, Istanbul 34810, Türkiye; sultan.buyuker@medipol.edu.tr; 2 Department of Pathology, Lady Reading Hospital, Peshawar 25000, Pakistan; khizar.abdullah@lrh.edu.pk; 3 Centre for Omics Sciences, Islamia College University, Peshawar 25120, Pakistan; abdulqadeerkhankhalil@gmail.com; 4 Animal Biotechnology Division, National Institute of Animal Science, RDA, 1500 Kongjwipatjwi-ro, Wanju-gun 55365, Republic of Korea; imrangnu@gmail.com; 5 Department of Pharmacy, University of Peshawar, Peshawar 25120, Pakistan; shahjahan.1504@outlook.com; 6 The National Research and Development Center for Sustainable Agriculture (Estidamah), Riyadh Technology Valley, Riyadh 12373, Saudi Arabia; adilm@estidamah.gov.sa (M.A.); aalkhamees@estidamah.gov.sa (A.H.A.-K.); 7 Department of Biotechnology, Yeungnam University, Gyeongsan 38541, Republic of Korea

**Keywords:** *Elaeocarpus*, *Elaeocarpus sylvestris*, phytochemicals, geraniin, mogrosides, pharmacological activity

## Abstract

*Elaeocarpus sylvestris* (Lour.) Poir., an evergreen tree native to East and Southeast Asia, has gained increasing scientific attention owing to its broad pharmacological properties. Traditionally used in East Asian medicine to treat inflammation, fever, and infectious diseases, modern research has revealed diverse bioactivities, including potent antioxidant, anti-inflammatory, antiviral, anticancer, antidiabetic, and immunomodulatory effects. This therapeutic potential is primarily attributed to its rich phytochemical composition, particularly polyphenols such as geraniin, 1,2,3,4,6-penta-*O*-galloyl-*β*-D-glucose and quercetin. This review particularly focuses on the chemistry of *E. sylvestris*, summarizing structurally elucidated compounds, including hydrolysable tannins, flavonoids, and triterpenoids, along with recent insights into the structure–activity relationships that underpin these antiviral, antioxidant, and anticancer activities. Recent studies have demonstrated substantial antiviral efficacy of *E. sylvestris* extracts and isolated compounds against major human pathogens, including herpesviruses, influenza A virus, and SARS-CoV-2, supported by in silico, in vitro, in vivo, and early-phase clinical evaluations. Its cosmeceutical applications, including antioxidant, skin-whitening, and blue-light protective effects, further highlight its multifunctional potential. To our knowledge, this is the first comprehensive review summarizing the phytochemistry, pharmacological activities, therapeutic potential, and cosmeceutical applications of *E. sylvestris*. Despite these promising findings, challenges remain in elucidating precise molecular mechanisms, pharmacokinetics, and clinical validation. This review identifies current research gaps and future directions necessary to advance *E. sylvestris* as a scientifically validated natural therapeutic resource.

## 1. Introduction

Medicinal plants have long served as a cornerstone of traditional and modern healthcare systems, providing a vast reservoir of bioactive compounds with diverse pharmacological potential [1]. Their therapeutic efficacy stems from complex mixtures of phytochemicals, including alkaloids, flavonoids, terpenoids, tannins, and phenolic acids, that interact synergistically to modulate multiple biological targets [2]. Recently, growing concerns over drug resistance, the side effects of synthetic medications, and the global demand for safer, natural remedies have intensified interest in plant-derived therapeutics [3]. Advances in analytical, molecular, and computational techniques have further facilitated the identification of novel lead compounds and the elucidation of their mechanisms of action [4]. Consequently, the systematic exploration of medicinal plants is essential not only for the discovery of new drugs but also for the scientific validation of traditional medicinal knowledge, thereby supporting their integration into evidence-based pharmacotherapy and functional health products [5].

Natural products have played a pivotal role in drug discovery and development for decades. Plants, or structurally modified derivatives of their constituents, account for the majority of clinically approved drugs [6,7]. Bioactive plant metabolites also exhibit antioxidant, antibacterial, antiviral, and anticancer activities [8,9,10,11,12]. For example, paclitaxel, derived from *Taxus brevifolia*, and camptothecin, isolated from *Camptotheca acuminata,* have improved cancer treatment and inspired the development of new therapeutic agents [13].

*Elaeocarpus* is a genus in the family Elaeocarpaceae, with many species distributed across tropical and subtropical regions [14,15]. The genus *Elaeocarpus* originates from the Greek words Elaeo (olive) and carpus (fruit), indicating that *Elaeocarpus* produces olive-like fruits [16]. *Elaeocarpus sylvestris* var. *ellipticus* (commonly known as Dampalsu) is of particular interest because of its distinctive phytochemical profile and ethnomedicinal applications. Its major constituents include tannin-type compounds such as geraniin and 1,2,3,4,6-penta-*O*-galloyl-*β*-D-glucose [17,18]. Reported biological activities of these compounds include antiviral effects against varicella-zoster and influenza A viruses, anti-inflammatory activity, radioprotective effects, and antioxidant properties [15]. Despite these promising findings, *E. sylvestris* remains relatively underexplored in the scientific literature. This review addresses this gap by synthesizing current knowledge on the chemical composition, biological activities, and potential therapeutic applications of *E. sylvestris*.

## 2. Literature Search Strategy

A comprehensive literature search was conducted to collect relevant studies on *Elaeocarpus sylvestris*. Electronic databases including PubMed, Scopus, Web of Science, and Google Scholar were systematically searched using relevant keywords such as “*Elaeocarpus sylvestris*”, “phytochemistry”, “secondary metabolites”, “pharmacological activities”, “antioxidant”, “antiviral”, “anticancer”, “antibacterial”, “antifungal” and “cosmeceuticals”, etc.

Only peer-reviewed articles published were considered. Studies were included based on their relevance to the phytochemical composition and biological activities of *E. sylvestris*, while unrelated, duplicate, or non-scientific reports were excluded. In addition, the chemical structures of the compounds were verified using the PubChem database and the chemical structures were constructed using SMILES (simplified molecular input line entry system) strings retrieved from Pubchem and visualized using ChemDraw software (23.1.2 32-bit version).

## 3. Global Distribution

**Figure 1:**
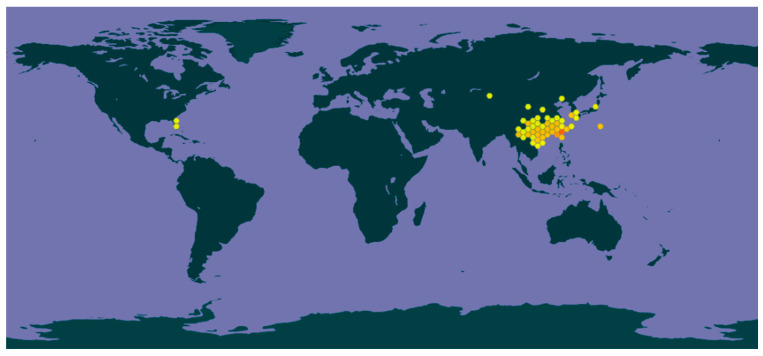
Global distribution of *Elaeocarpus sylvestris*. Map downloaded from GBIF (GBIF.org, accessed 10 October 2025; https://www.gbif.org/species/7777372), based on occurrence records available in database.

*Elaeocarpus sylvestris* is native to East and Southeast Asia and exhibits a preference for warm-temperate to subtropical evergreen forests. In China, it is widely distributed across southern and central provinces, including Fujian, Guangdong, Guangxi, Guizhou, Hainan, Hunan, Jiangxi, Sichuan, Yunnan, and Zhejiang, typically at elevations between 300 and 2000 m. Its range also extends to Vietnam, Taiwan, Japan, and Korea, where it inhabits similar forest environments (Figure 1) [19]. In Korea, *E. sylvestris* is mainly distributed in the southern regions of Jeju Island, where it thrives under mild climatic conditions. In Japan, it is widespread in temperate forests, particularly on the southern islands. This distribution pattern reflects its sensitivity to low temperatures, which limits its presence to warmer regions of its global range [20].

## 4. Botanical and Ethnomedicinal Background

*Elaeocarpus sylvestris* (Lour.) Poir., a member of the family Elaeocarpaceae, is an evergreen tree widely distributed across East Asia. The species was originally described as *Adenodus sylvestris* by Loureiro in 1790 and was later reclassified under the genus *Elaeocarpus*. Several synonyms are documented in botanical literature, including *E. henryi* Hance, *E. kwangtungensis* Hu, and *E. omeiensis* Rehder & E. H. Wilson (POWO, accessed 3 October 2025).

**Figure 2:**
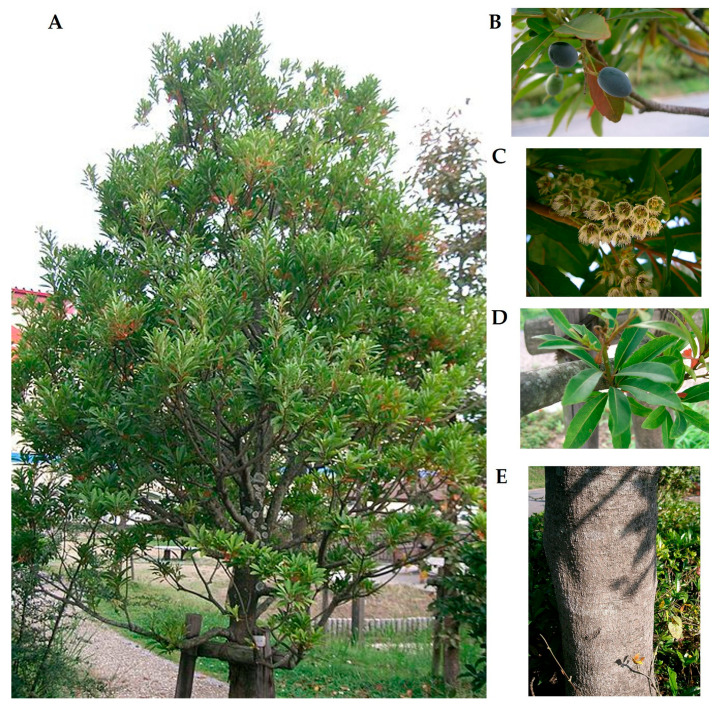
Morphological features of *Elaeocarpus sylvestris* (Lour.) Poir.: (**A**) whole plant habit; (**B**) fruits; (**C**) flowers; (**D**) leaves; (**E**) trunk. Images adapted from Wikimedia Commons (KENPEI), licensed under CC BY-SA 4.0.

This species typically reaches about 15 m in height. It has slender branchlets that are sparsely covered with pale hairs when young. The leaves are obovate to oblanceolate, measuring 4–12 × 2–7 cm, papery in texture, and glabrous on both surfaces. When dried, the foliage often appears blackish brown. The venation is characterized by 4–5 pairs of lateral veins, which are conspicuous on the abaxial surface but faint on the adaxial side. Veinlets are relatively sparse but evident on the upper surface. The leaf base is narrowly cuneate and decurrent, the margins are crenate to sinuately crenate, and the apex is obtuse to shortly acuminate (Figure 2). The petiole measures 1–1.5 cm in length, being initially pubescent but becoming glabrous with maturity [19].

Inflorescences are arranged as racemes, typically 4–6 cm long, and borne in the axils of fallen or current leaves. The peduncles are slender and glabrous, though sometimes sparsely covered with gray hairs. The pedicels measure 3–5 mm and are usually glabrous. The flowers are bisexual, with five lanceolate sepals (ca. 4 mm) that are sparsely pubescent on both surfaces and five obovate petals that are deeply laciniate in their upper half, forming 10–12 linear lobes. There are about 15 stamens, each approximately 3 mm long, with puberulent anthers lacking apical awns. The floral disk is five-lobed, globose, and distinctly white-pubescent. The ovary is pubescent, two- or three-loculed, with a style about 2 mm long that is pubescent in its lower half (Figure 2) [19].

The fruit is an ellipsoid drupe measuring 1–1.2 × ~0.7 cm, with a thin, bony endocarp that has three ventral sutures. Flowering occurs from April to May, and fruiting takes place between May and August. Ecologically, the species thrives in evergreen forests at elevations of 300 to 2000 m, particularly in southern and central China, including the provinces of Fujian, Guangdong, Guangxi, Guizhou, Hainan, Hunan, Jiangxi, Sichuan, Yunnan, and Zhejiang. Its distribution also extends into Vietnam [19].

*Elaeocarpus sylvestris* is traditionally used to treat anxiety, asthma, arthritis, stress, depression, palpitations, neuralgia, epilepsy, migraine, hypertension, liver diseases, diabetes, and malaria [21]. Nevertheless, there is a lack of primary ethnobotanical documentation, and there is very little information on the parts of plants used or how they were prepared. Generally, related species are given in decoction or infusion, alone or in combination with other herbs. Very little is recorded of its application in Traditional Chinese Medicine, or Korean, Japanese or Taiwanese traditional medicine, indicating that its ethnomedicinal use is underreported.

## 5. Phytochemical Constituents

To date, 41 chemical constituents have been identified from *E. sylvestris* (Table 1). These compounds are classified into five major groups: tannins, phenolics, flavonoids, sterols and triterpenoids, and other miscellaneous compounds. Collectively, these phytochemicals contribute to the species’ broad pharmacological potential, including anticancer, antioxidant, anti-inflammatory, antiviral, antibacterial, and hepatoprotective activities.

### 5.1. Tannins

**Figure 3:**
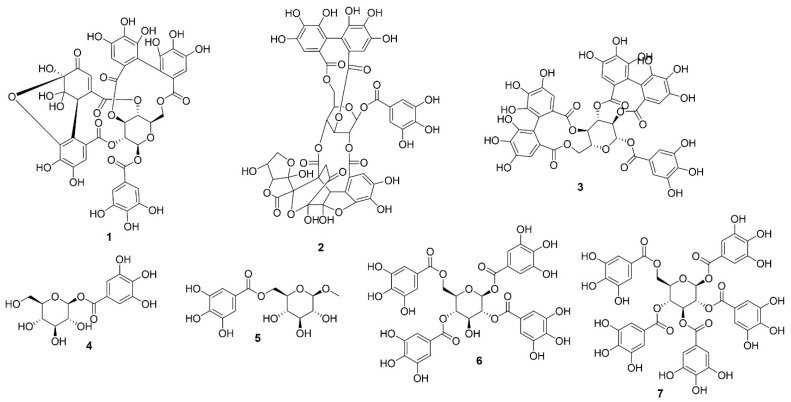
Structures of compounds **1**–**7**.

Tannins are high-molecular-weight polyphenolic compounds widely distributed in plants and known for their ability to form complexes with proteins and other macromolecules [22]. *Elaeocarpus sylvestris* contains several hydrolysable tannins, including geraniin, elaeocarpusin, casuarictin, and various galloyl-glucose derivatives such as mono-, tetra-, and penta-galloylglucose (Compounds **1**–**7**) (Figure 3; Table 1).

### 5.2. Phenolic Acids and Their Esters

**Figure 4:**
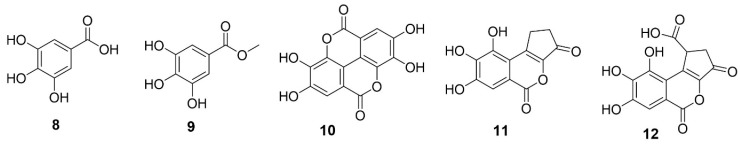
Structures of compounds **8**–**12**.

Phenolic compounds are plant-derived secondary metabolites characterized by one or more hydroxyl groups attached to aromatic rings and are widely distributed across diverse plant species [23]. In *E. sylvestris*, gallic acid, methyl gallate, ellagic acid, brevifolin, and brevifolin carboxylic acid have been reported (Compounds **8**–**12**) (Figure 4; Table 1).

### 5.3. Flavonoids

**Figure 5:**
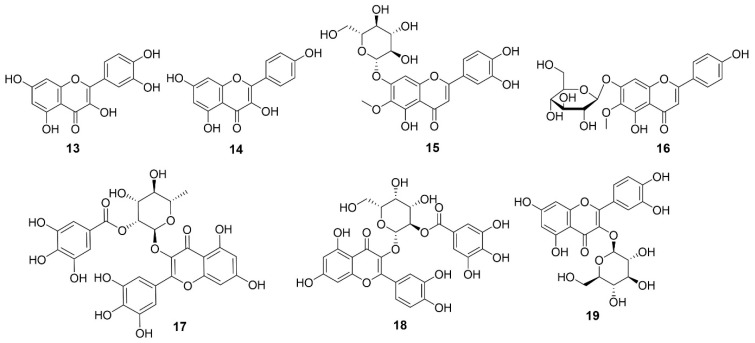
Structures of compounds **13**–**19**.

Flavonoids are a diverse group of polyphenolic metabolites characterized by a C6–C3–C6 carbon skeleton [24,25]. In *E. sylvestris*, several flavonoids and their glycosides have been identified, including quercetin, kaempferol, nepitrin, homoplantaginin, myricitrin 2′′-*O*-gallate, and 2′′-*O*-galloylhyperin (Compounds **13**–**19**) (Figure 5; Table 1).

### 5.4. Sterols and Triterpenoids

**Figure 6:**
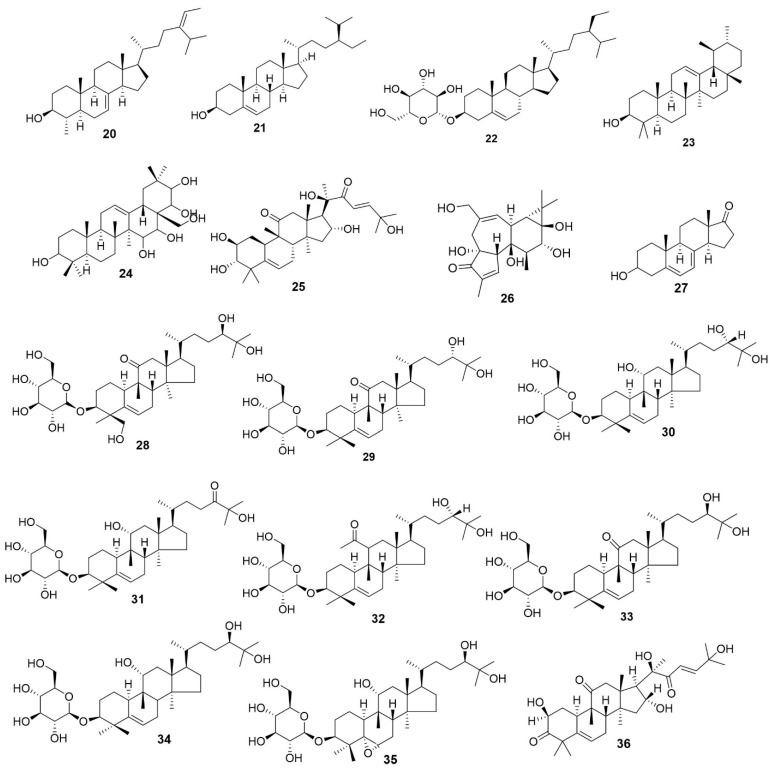
Structures of compounds **20**–**36**.

Sterols and triterpenoids are important classes of plant secondary metabolites biosynthesized from isoprene units via the mevalonate pathway [26]. In *E. sylvestris*, several sterols and terpenoids have been identified, including *α*- and *β*-sitosterol, daucosterol, α-amyrin, cucurbitacins D and F, and oleanane-type derivatives such as 3,15,16,21,22,28-hexhydroxyl-12-oleanene. Additionally, several mogrosides, such as I E1, I E2, epimogrosides, and their oxo- or acetylated derivatives, have been reported (Compounds **20**–**36**) (Figure 6; Table 1).

### 5.5. Other Compounds

**Figure 7:**
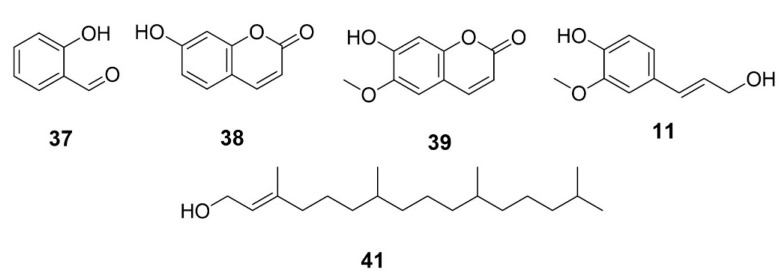
Structures of compounds **37**–**41**.

In addition to polyphenols and terpenoids, *E. sylvestris* contains simpler aromatic and coumarin-type metabolites, including 2-hydroxybenzaldehyde, coniferyl alcohol, umbelliferone, and scopoletin (Compounds **37**–**41**) (Figure 7; Table 1). Although extensive phytochemical studies have been conducted on *E. sylvestris*, there are currently no reports describing the presence or characterization of fixed or essential oils in this species. In contrast, seed-derived fixed oils with potential cosmetic and industrial applications have been reported in related species such as *E*. *serratus*. This indicates that lipid constituents in *E. sylvestris* remain an unexplored area that warrants further investigation [27].

**Table 1:** Chemical constituents of *E. sylvestris*.

S/N	Compound Name/Class	Reference
**Tannin**		
**1**	Geraniin	[18]
**2**	Elaeocarpusin	[18]
**3**	Casuarictin	[28]
**4**	1-*O*-galloyl-*β*-D-glucose (*β*-glucogallin)	[18]
**5**	Methyl 6-*O*-galloyl-*β*-D-glucopyranoside	[18]
**6**	1,2,4,6-Tetra-*O*-galloyl-*β*-D-glucose	[29]
**7**	1,2,3,4,6-penta-*O*-galloyl-*β*-D-glucose	[17,29]
**Phenolic acids and their esters**		
**8**	Gallic acid	[20]
**9**	Methyl gallate	[30]
**10**	Ellagic acid	[29,31]
**11**	Brevifolin	[29,31]
**12**	Brevifolin carboxylic acid	[29]
**Flavonoid**		
**13**	Quercetin	[31,32]
**14**	Kaempferol	[31,32]
**15**	Nepitrin	[29]
**16**	Homoplantaginin	[29]
**17**	Myricitrin 2′′-*O*-gallate	[29]
**18**	2′′-*O*-galloylhyperin	[29]
**19**	Isoquercitrin	[32]
**Sterol/Triterpenoid**		
**20**	*α*-Sitosterol	[33]
**21**	*β*-Sitosterol	[34]
**22**	Daucosterol	[34]
**23**	*α*-Amyrin	[33]
**24**	3,15,16,21,22,28-Hexhydroxyl-12-oleanene	[33]
**25**	Cucurbitacin F	[35]
**26**	Phorbol	[33]
**27**	3-hydroxyl-androst-5,7-dien-17-one	[33]
**28**	29-hydroxymogroside I E2	[36]
**29**	epimogroside I E2	[36]
**30**	epimogroside I E1	[36]
**31**	24-oxomogroside I E1	[36]
**32**	11-O-acetylmogroside I E1	[36]
**33**	Mogroside I E2	[36]
**34**	Mogroside I E1	[36]
**35**	5α,6α-epoxymogroside I E1	[36]
**36**	Cucurbitacin D	[36]
**Other Compounds**		
**37**	2-Hydroxy-benzaldehyde	[34]
**38**	Umbelliferone	[34]
**39**	Scopoletin	[34]
**40**	Coniferyl alcohol	[34]
**41**	3,7,11,15-Tetramethyl-2-hexadecen-1-ol	[33]

The bioactive metabolites of *E. sylvestris* exhibit distinct structural features such as multiple galloyl moieties in hydrolysable tannins, C3–C4 double bonds and acetyl substitutions in cucurbitane-type triterpenoids, and hydroxylation or glycosylation patterns in flavonoids. These chemical modifications are directly linked to their antioxidant, cytotoxic, and antiviral activities, highlighting the critical role of structure–activity relationships (SAR) in their pharmacological profiles.

## 6. Pharmacological Activities

**Figure 8:**
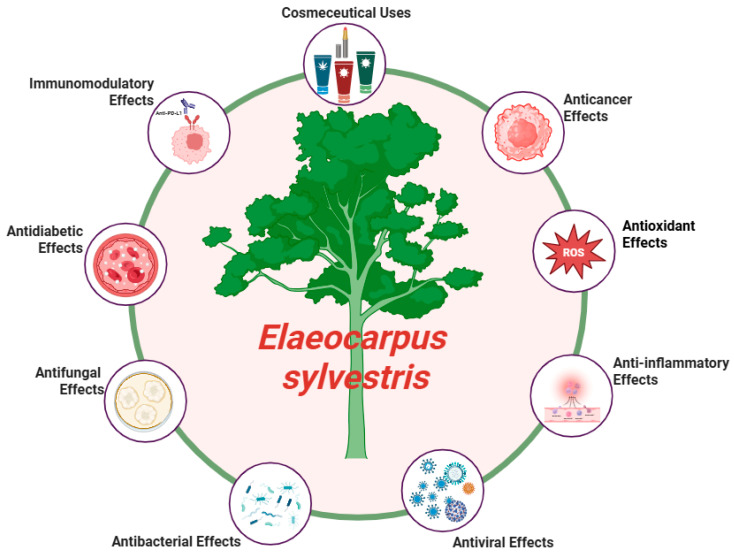
Pharmacological activities of *E. sylvestris*. Created with BioRender.com.

Previous studies have reported that *E. sylvestris* exhibits diverse biological activities (Figure 8). These include anticancer, antioxidant, anti-inflammatory, antiviral, antibacterial, antifungal, antidiabetic, and immunomodulatory effects.

### 6.1. Anticancer Effects

Cancer is a multifactorial disease resulting from genetic mutations that cause uncontrolled cell growth and malignant transformation [37]. Although conventional treatments such as chemotherapy and radiotherapy remain standard, their adverse side effects often limit long-term efficacy [38]. Natural products offer a promising source of safer and more effective anticancer compounds owing to their ability to modulate key cellular and molecular pathways [39].

**Figure 9:**
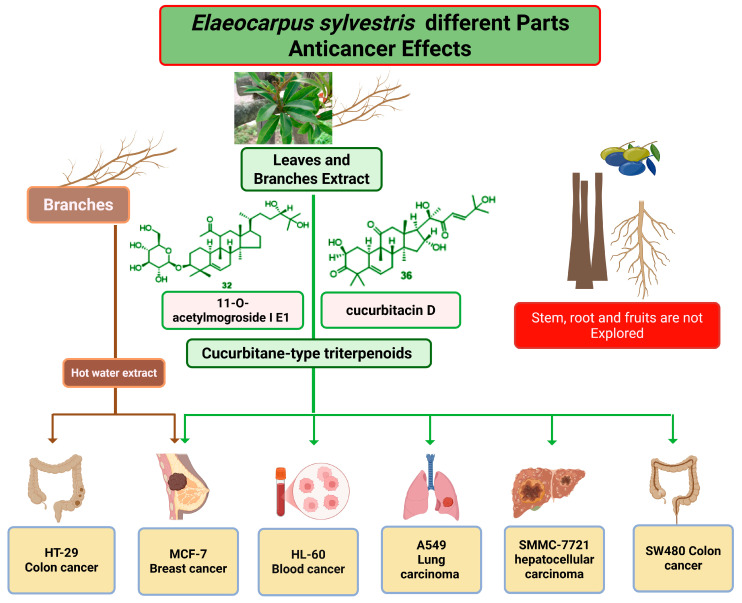
Anticancer effects of *E. sylvestris* extracts and compounds on different cell lines. Created with BioRender.com.

Several studies have demonstrated the anticancer potential of *E. sylvestris* through its phytochemical constituents and extracts (Figure 9; Table 2). Hot water extracts of *E. sylvestris* exhibited cytotoxic activity against multiple cancer cell lines, including breast (MCF-7) and colon (HT-29) cancer cells. In comparative assays, *E. sylvestris* extracts (ESE) demonstrated stronger inhibition of cell proliferation than *Magnolia officinalis*, indicating superior anticancer efficacy. At concentrations of 1–10 mg/mL, ESE significantly suppressed proliferation in MCF-7 and HT-29 cells [40]. Similarly, yeast-fermented extracts of *E. sylvestris* displayed cytotoxicity against HT-29, MCF-7, and INT407 cells while simultaneously reducing proinflammatory cytokines, suggesting combined anti-inflammatory and anticancer benefits [41].

In addition to crude extracts, purified secondary metabolites of *E. sylvestris* have been identified with potent anticancer activities. Wang et al. (2022) reported the isolation of nine cucurbitane-type triterpenoids from its branches and leaves, among which cucurbitacin D (**36**) and 11-*O*-acetylmogroside I E1 (**32**) exhibited notable cytotoxicity against human cancer cell lines, including HL-60 leukemia, A549 lung adenocarcinoma, SMMC-7721 hepatoma, MCF-7 breast cancer, and SW480 colon cancer [36]. These findings highlight the structural relevance of functional groups, such as 11-*O*-acetyl, in enhancing cytotoxicity. Furthermore, methanolic extracts of *E. sylvestris* var. *ellipticus* displayed cytotoxic effects in fibroblast models alongside antimicrobial activity, suggesting broader biomedical applications beyond anticancer research [42].

Beyond direct cytotoxicity, *E. sylvestris* exhibits protective effects against apoptosis in radiation-induced damage models. Park et al. (2011) demonstrated that extracts rich in 1,2,3,4,6-penta-*O*-galloyl-*β*-D-glucose (**7**) protected lymphocytes and intestinal crypt cells from radiation-induced apoptosis in vivo, thereby enhancing radioresistance [43]. This radioprotective role, combined with its cytotoxic activity, underscores the dual potential of *E. sylvestris* in cancer therapy as both a direct anticancer agent and an adjuvant capable of mitigating treatment-related side effects. Overall, the anticancer potential of *E. sylvestris* is largely associated with its structurally diverse triterpenoids and ellagitannins, where acetylated cucurbitane skeletons and multiple galloyl groups enhance cytotoxic and pro-apoptotic activity.

### 6.2. Antioxidant Effects

Oxidative stress results from the overproduction of reactive oxygen species (ROS), which can overwhelm the body’s endogenous antioxidant defenses and cause cellular and molecular damage [44]. Such oxidative imbalances play a critical role in the onset and progression of various pathological conditions, including inflammation, aging, cancer, and metabolic disorders [45,46]. Natural antioxidants from medicinal plants have demonstrated strong potential in neutralizing ROS, thereby protecting biomolecules and maintaining cellular homeostasis [47].

*Elaeocarpus sylvestris* has been extensively studied for its potent antioxidant properties, which are attributed to its rich content of polyphenols, flavonoids, and other bioactive compounds. Both crude extracts and purified metabolites have demonstrated significant radical scavenging activities in vitro and in cell-based assays (Table 3) [15]. For example, 1,2,3,4,6-penta-*O*-galloyl-*β*-D-glucose (**7**), isolated from *E. sylvestris* var. *ellipticus*, effectively scavenged DPPH radicals and intracellular ROS, restored antioxidant enzyme activities, including those of superoxide dismutase, catalase, and glutathione peroxidase, and inhibited lipid peroxidation in H_2_O_2_-treated cells [17]. Similarly, methanolic leaf extracts exhibited remarkable DPPH scavenging activity, with IC_50_ values as low as 1.86 μg/mL, indicating strong free radical neutralization capacity [48].

Endophytic fungi associated with *E. sylvestris* also contribute to its antioxidant potential. *Pseudocercospora* sp. ESL 02, isolated from leaves and stems, produced compounds such as terreic acid and 6-methylsalicylic acid, exhibiting potent DPPH radical scavenging activity (IC_50_: 0.22 and 3.87 mmol/L, respectively) and strong reducing power and *β*-carotene bleaching activity [49]. Furthermore, biotechnological approaches, including yeast and lactic acid bacteria fermentation of hot water extracts, significantly enhanced the antioxidant capacity of *E. sylvestris*, as evidenced by increased DPPH and ABTS radical scavenging activities and changes in the metabolomic profiles of carboxylic acids and polyphenols [41,50].

Nanotechnology-based formulations have further expanded the antioxidant potential of *E. sylvestris*. Leaf extract-capped copper oxide nanoparticles and magnetite nanoparticles (ESMNPs) synthesized using ESE demonstrated strong radical scavenging abilities, providing sustainable approaches for bioactive delivery and therapeutic applications [51,52]. Comparative studies indicate that branch and leaf extracts of *E. sylvestris* exhibit higher antioxidant activity than those of other subtropical plants, including *Magnolia officinalis*, highlighting its potential as a promising natural antioxidant source for food, cosmetic, and pharmaceutical industries [20,31,40]. In summary, antioxidant properties mainly derive from polyphenols such as geraniin (**1**) and penta-*O*-galloyl-*β*-D-glucose, whose multiple hydroxyl groups confer high radical-scavenging and metal-chelating capacities.

### 6.3. Anti-Inflammatory Effects

Inflammation is a complex biological response to injury or infection; its chronic activation contributes to numerous disorders, including arthritis, diabetes, and cardiovascular diseases [53]. Although synthetic anti-inflammatory drugs are effective, their prolonged use often leads to adverse effects [54]. Natural products offer safer alternatives, as their bioactive compounds can modulate inflammatory pathways and restore immune balance [25,55].

**Figure 10:**
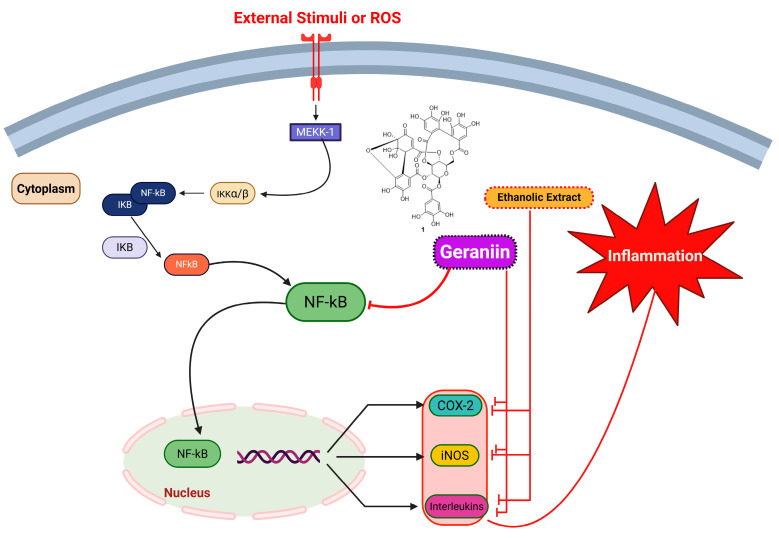
Proposed anti-inflammatory mechanism of *E. sylvestris* extract and its bioactive compound via inhibition of NF-κB signaling pathways. Created with BioRender.com.

*Elaeocarpus sylvestris* exhibits substantial anti-inflammatory properties, as demonstrated in both in vitro and in vivo models (Figure 10; Table 4). In rheumatoid arthritis models, ESE suppressed lipopolysaccharide-induced nitric oxide and proinflammatory cytokine production in RAW264.7 macrophages. Co-administering ESE with sulfasalazine in collagen-induced arthritis mice reduced arthritis scores, joint edema, and serum levels of inflammatory cytokines and downregulated chemokine expression, suggesting a synergistic effect in improving arthritis treatment outcomes [56].

Fermentation of hot water extracts of *E. sylvestris* var. *ellipticus* with lactic acid bacteria (*Lactobacillus kimchicus* or *L. plantarum*) further enhanced its anti-inflammatory potential. Fermented extracts markedly reduced the expression of pro-inflammatory cytokines IL-6 and TNF-*α*, likely via modulation of the NF-κB signaling pathway. These findings highlight the ability of fermentation to enrich bioactive compounds, such as polyphenols and organic acids, which contribute to anti-inflammatory effects [50]. Additionally, the isolated triterpenoid elaeocarpusin (**2**) demonstrated potent inhibition of mast cell-mediated allergic inflammation. Elaeocarpusin (**2**) suppressed mast cell degranulation markers, including histamine and *β*-hexosaminidase, reduced intracellular calcium levels, and reduced TNF-α and IL-4 expression. In vivo, elaeocarpusin (**2**) attenuated IgE-mediated passive cutaneous anaphylaxis and ovalbumin-induced systemic anaphylaxis, indicating its therapeutic potential for allergic and inflammatory diseases [57]. Overall, the anti-inflammatory activity of *E. sylvestris* is primarily attributed to its polyphenolic and ellagitannin constituents, such as geraniin (**1**) and elaeocarpusin (**2**), which exert potent inhibitory effects on the NF-κB and MAPK signaling pathways. These structural features, particularly multiple hydroxyl and galloyl groups, enable effective downregulation of pro-inflammatory mediators (iNOS, COX-2, TNF-α, IL-1β, IL-6) and suppression of mast cell degranulation, contributing to both anti-arthritic and antiallergic effects in vitro and in vivo.

### 6.4. Antiviral Effects

Viral infections pose a major global health threat, causing considerable morbidity and mortality [58]. Natural products constitute the most important source of antiviral agents for treating viral infections [59].

**Figure 11:**
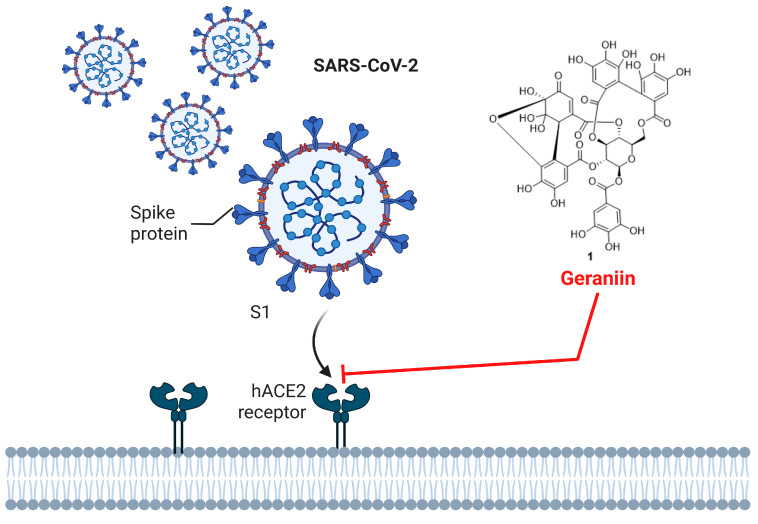
Schematic diagram illustrating the blockade of SARS-CoV-2 spike protein–hACE2 receptor interaction by geraniin. Created with BioRender.com.

ESE has demonstrated broad-spectrum antiviral activity against several human viral pathogens, including varicella-zoster virus (VZV), human cytomegalovirus (HCMV), herpes simplex virus (HSV), influenza A virus (IAV), and SARS-CoV-2 (Figure 11; Table 5). Ethanol extracts and their ethyl acetate fractions markedly inhibited viral replication in vitro by suppressing immediate-early (IE) and lytic gene expression, including VZV IE62, HCMV major IE, and HSV ICP0, gD, and gB genes, without exhibiting considerable cytotoxicity in host cells [21,28,32,60,61,62,63]. Ethanolic extracts and bioactive compounds, including 1,2,3,4,6-penta-*O*-galloyl-*β*-D-glucose (**7**), geraniin (**1**), quercetin (**13**), isoquercitrin (**19**), methyl gallate (**9**), and casuarictin (**3**), were identified as major contributors to the antiviral effects of *E. sylvestris*. These constituents act through mechanisms that include inhibition of viral gene expression, blockade of viral entry, and promotion of autophagy-mediated viral degradation [28,32,60,61,62,63,64].

ESE has also exhibited substantial antiviral efficacy in animal models, corroborating its in vitro efficacy. Oral administration of ESE or its bioactive components reduced viral load, reduced lung lesions, alleviated inflammatory responses, and improved survival in models of VZV, HSV-1, IAV, and SARS-CoV-2 infections [28,61,62,65,66]. In SARS-CoV-2-infected Syrian hamsters, ESE decreased viral replication, reduced fever and body weight loss, and improved lung pathophysiology [66]. Similarly, BALB/c nude mice infected with HSV-1 or VZV exhibited reduced viral proliferation and virus-induced pain following treatment with ESE or geraniin (**1**) [28,61]. In addition, screening studies revealed that ESE exhibited virucidal activity against murine norovirus, a surrogate for human norovirus, indicating antiviral potential beyond herpesviruses [67]. Collectively, these findings indicate that ESE can modulate host–pathogen interactions and exert systemic antiviral and anti-inflammatory effects in vivo.

Clinical investigations support the safety and therapeutic potential of *E. sylvestris* and its formulations in humans. ES16001, an ethanol extract of *E. sylvestris* leaves, was well tolerated in single and multiple ascending dose trials, with only mild adverse effects observed [68]. Moreover, clinical trials in patients with mild COVID-19 demonstrated accelerated recovery, reduced inflammatory mediator levels, and improved psychological well-being [66]. Together, these preclinical and clinical data support the use of ESE as a natural antiviral agent, with mechanistic actions targeting viral replication, entry, and virus-induced inflammation [28,32,61,62,66,69]. The antiviral potential of *E. sylvestris* has been attributed to its diverse secondary metabolites, particularly polyphenols, ellagitannins, and galloyl derivatives such as geraniin (**1**), methyl gallate, casuarictin, and 1,2,3,4,6-penta-*O*-galloyl-*β*-D-glucose. These compounds exhibit multitarget mechanisms, including inhibition of viral entry, suppression of immediate-early (IE) gene expression, and blockade of replication-associated pathways across various viruses such as VZV, HSV, HCMV, IAV, and SARS-CoV-2. The strong antiviral efficacy is closely linked to structural features like multiple hydroxyl and galloyl groups, which enhance viral protein binding and disrupt replication signaling.

**Table 5:** Antiviral effects of *E. sylvestris* extracts and compounds.

Compound/Extract	Source (Plant Part)	Target Virus	In Vitro/In Vivo	Mechanism of Action	Key Effects	Reference
1,2,3,4,6-penta-O-galloyl-β-D-glucose (**7**)	Leaves (EtOAc fraction)	Varicella-zoster virus (VZV), influenza A virus (IAV)	In vitro/In vivo	Suppresses viral IE gene expression, inhibits viral RNA/protein production, blocks multiple replication steps	Reduced viral replication, viral RNA/protein levels, and disease symptoms in mice	[60,65]
Geraniin (**1**)	Leaves, fruits	Herpes simplex virus (HSV-1), SARS-CoV-2, IAV	In vitro/In vivo	Blocks viral entry by interfering with spike-ACE2 binding (SARS-CoV-2), inhibits viral replication, reduces ICP0 expression (HSV-1)	Reduced viral proliferation, lung lesions, and mortality in mice and hamsters	[28,64,65]
Quercetin (**13**)	Leaves (EtOAc fraction)	VZV, human cytomegalovirus (HCMV)	In vitro	Inhibits immediate-early gene expression	Suppressed viral replication without cytotoxicity	[32]
Isoquercitrin (**19**)	Leaves (EtOAc fraction)	VZV, HCMV	In vitro	Inhibits immediate-early gene expression	Suppressed viral replication	[32]
Methyl gallate	Fruits, leaves, stems	HSV-1, HSV-2, VZV	In vitro/In vivo	Suppresses viral replication genes, enhances autophagy	Reduced viral replication, alleviated pain in mice, no toxicity	[28]
Casuarictin (**3**)	Fruits, leaves, stems	HSV-1, HSV-2, VZV	In vitro/In vivo	Suppresses viral replication genes, enhances autophagy	Reduced viral proliferation, prolonged survival in mice	[28]
Ethyl acetate	Leaves	HCMV	In vitro	Inhibits HCMV major immediate-early enhancer/promoter activity; reduces lytic gene expression	Most active fraction; significantly lowered HCMV replication without cytotoxicity	[69]
Ethanol extract	Leaves, stems, fruits	VZV, HCMV, HSV-1, HSV-2, IAV, SARS-CoV-2	In vitro/In vivo/Clinical	Inhibits viral gene expression, replication, viral entry, and virus-induced inflammation	Reduced viral load, inflammation, lung lesions, pain; accelerated recovery in humans	[28,61,62,66,70]
Ethanol extract	Leaves	HCMV	In vitro	Inhibits SARS-CoV-2 replication; proposed for prevention and treatment	Demonstrated inhibitory effect on viral replication; patented as a therapeutic agent	[63]
Ethanol extract	Standardized clinical formulation	VZV	Human clinical (SAD/MAD)	Inhibits VZV reactivation; modulates host–virus signaling	Well tolerated in healthy adults (n = 48); only mild reversible ALT elevations; no serious adverse events	[68]
*(Screening context)* Korean endemic plant extract set (including *E. sylvestris*)	Leaves	Murine norovirus (surrogate of human norovirus)	In vitro	General virucidal screening of 28 Korean endemic plant extracts	Only limited inhibition observed; study context supports broad antiviral exploration	[67]

### 6.5. Antibacterial Effects

*Elaeocarpus sylvestris* exhibits potent antibacterial activity attributable to its rich polyphenolic and flavonoid content (Table 6). Early studies reported that prodelphinidin oligomers isolated from the bark of *E. sylvestris var. ellipticus* exhibited potent antibacterial activity, with low minimum inhibitory concentrations (98–389 µg/mL) against *Staphylococcus aureus* and *Vibrio* species [71]. The presence of 3,4,5-trihydroxyphenyl groups was considered critical for their strong antibacterial activity. Methanolic leaf extracts of *E. sylvestris* also displayed pronounced inhibition against *Helicobacter pylori*, producing inhibition zones ≥ 20 mm at 1 mg/disk, comparable to standard antibiotics such as amoxicillin and tetracycline, supporting its potential as a natural anti-*H. pylori* agent [72].

Further investigations on evergreen woody plants from Jeju Island confirmed *E. sylvestris var. ellipticus* as one of the most potent species against *S. aureus*. Methanolic leaf extracts produced inhibition zones up to 23.3 mm, exceeding those of synthetic antimicrobials such as methylparaben and phenoxyethanol, and the n-butanol fraction retained similar potency [73,74]. Additionally, ethanolic and methanolic extracts demonstrated inhibitory zones of 20 to 23 mm against *Trichophyton mentagrophytes*, suggesting antifungal and antibacterial effects [75]. Recent studies have focused on nanotechnology-based formulations, where *E. sylvestris* leaf extract served as a reducing and capping agent in the green synthesis of ZnO and Fe_3_O_4_ nanoparticles. These bioengineered nanoparticles demonstrated substantial antibacterial activity against *Staphylococcus aureus*, *Escherichia coli*, *Pseudomonas aeruginosa*, and *Enterococcus faecalis* [52,76].

Moreover, methanolic extracts of *E. sylvestris* leaves and branches completely inhibited the growth of *S. aureus* and *Listeria monocytogenes* at 100 mg/mL and reduced lactic acid bacteria and oxidative stress in vitro [42,77]. These findings highlight the multifunctional bioactivities of *E. sylvestris*, combining antimicrobial, antioxidant, and cytoprotective properties. Collectively, both crude extracts and nanoparticle formulations of *E. sylvestris* are promising candidates for use in food preservation, pharmaceuticals, and cosmetics as natural antibacterial and bioactive agents.

### 6.6. Antifungal Effects

*Elaeocarpus sylvestris* exhibits notable antifungal potential, as demonstrated in laboratory and applied studies. ESMNPs displayed remarkable antifungal activity against *Aspergillus niger*, highlighting the utility of ESE in green nanotechnology applications [52]. In comparative studies of tree species, ESE displayed higher antifungal activity against *Fusarium graminearum* than several other woody plants, suggesting its effectiveness in controlling fungal pathogens in ecological and practical contexts [78]. Collectively, these findings indicate that *E. sylvestris* is a valuable source of antifungal agents for pharmaceutical, agricultural, and environmental applications.

### 6.7. Antidiabetic Effects

Studies highlight the antidiabetic potential of *E. sylvestris* through inhibition of carbohydrate-digesting enzymes. Methanol extracts of *E. sylvestris* var. *ellipticus* leaves demonstrated strong α-glucosidase inhibitory activity, with IC_50_ values ranging from 22 to 92 μg/mL. In addition, ethyl acetate and butanol extracts of the leaves substantially inhibited the effects of yeast α-glucosidase and rat intestinal sucrase, indicating a potential to regulate postprandial hyperglycemia by delaying carbohydrate breakdown [79]. These findings suggest that *E. sylvestris* is a promising natural source of α-glucosidase inhibitors for plant-based antidiabetic agents.

### 6.8. Immunomodulatory and Radioprotective Effects

*Elaeocarpus sylvestris* has demonstrated considerable immunomodulatory and radioprotective effects in vivo. In *γ*-ray irradiated mice, ESE enriched with 1,2,3,4,6-penta-*O*-galloyl-*β*-D-glucose (**7**) markedly improved survival by enhancing hematopoietic recovery and stimulating immune cell proliferation. Mice treated with the extract exhibited higher counts of endogenous colony-forming units, accelerated proliferation of lymphocytes and granulocytes, and restoration of spleen weight compared with untreated irradiated controls. Furthermore, splenocyte proliferation, measured via thymidine incorporation, was markedly elevated, indicating mitigation of radiation-induced immunosuppression through stimulation of hematopoietic and immune systems. These findings highlight the potential of *E. sylvestris* as a natural immunoprotective agent against radiation-induced damage [80].

### 6.9. Cosmeceutical Applications

*Elaeocarpus sylvestris* has gained increasing attention in cosmeceuticals owing to its potent antioxidant, antimicrobial, and skin-protective properties. Leaf and branch extracts exhibit strong free radical scavenging capacity and antimicrobial effects, including complete inhibition of *Staphylococcus aureus* and *Listeria monocytogenes*, while reducing H_2_O_2_ formation by over 50% at 100 mg/mL. Methanolic extracts demonstrate low cytotoxicity in human skin fibroblasts and contain several metabolites with potential roles in skin improvement and photoprotection, supporting their utility in functional skincare formulations [77]. Screening studies on Jeju Island medicinal plants further confirmed that *E. sylvestris* var. *ellipticus* possesses strong antioxidant activity (IC_50_: 13.99 μg/mL) and inhibits melanin synthesis in B16 F10 melanoma cells in a dose-dependent manner, indicating its potential as a natural anti-aging and skin-whitening agent [81].

ESE has also been incorporated into advanced cosmetic formulations for photoprotection. A patented composition combining *E. sylvestris* var. *ellipticus* with *Curcuma aromatica* effectively blocked harmful blue light (380–500 nm), thereby preventing light-induced skin damage [82]. Furthermore, comparative studies have revealed that subcritical water extracts of *E. sylvestris* leaves contain the highest gallic acid levels, correlating with superior antioxidant, anti-acne, and skin-whitening activities, establishing *E. sylvestris* as a low-toxicity natural whitening ingredient [20]. Collectively, these findings position *E. sylvestris* as a promising multifunctional component in next-generation skin care products. Notably, while cosmetic applications of plant-derived oils are well established, there are currently no reports on oil extraction or utilization from *E. sylvestris*. In contrast, related species such as *Elaeocarpus serratus* have demonstrated the presence of seed-derived fixed oils with potential use in cosmetic and industrial formulations [27]. This highlights an opportunity for future studies to explore lipid-based ingredients from *E. sylvestris* for cosmeceutical applications.

## 7. Structure–Activity Relationship

Structure–activity relationship (SAR) analysis provides insight into how specific structural features influence the biological activity [83]. Recent phytochemical investigations of *E. sylvestris* have revealed the presence of cucurbitane-type triterpenoids isolated from its branches and leaves, exhibiting notable cytotoxic and bioactive properties. The biological potency of these triterpenoids is strongly influenced by the number and position of hydroxyl, carbonyl, and double bond substitutions on the cucurbitane skeleton. In particular, compounds bearing hydroxyl substitutions at C-3, C-7, and C-23 positions showed enhanced cytotoxic and antiproliferative activities, suggesting that the introduction of polar groups increases interaction with biological targets. Conversely, acetylation or the absence of hydroxyl groups at these positions led to reduced bioactivity, emphasizing the importance of free hydroxyl functionalities for activity [36].

Furthermore, the presence of an unsaturated bond between C-5 and C-6 and a carbonyl group at C-11 appeared crucial for maintaining cytotoxic potency. Comparative analysis of analogs also indicated that modifications in the side chain length and degree of oxidation at C-17 significantly altered biological effects. Collectively, these findings demonstrate that minor structural modifications on the cucurbitane scaffold can dramatically influence the pharmacological properties of *E. sylvestris* constituents, providing valuable insights for designing semi-synthetic derivatives with improved efficacy and selectivity [36].

## 8. Toxicological and Safety Profile

Available toxicological evidence indicates that *Elaeocarpus sylvestris* (Lour.) Poir. extracts generally exhibit low toxicity, particularly in in vitro and short-term human studies. Several investigations have demonstrated selective antiviral and cytoprotective effects with minimal cytotoxicity across different cell models [50,61,69]. For example, leaf extracts and their fractions showed antiviral activity against herpes simplex virus and human cytomegalovirus without significant adverse effects on host cell viability, while isolated compounds such as geraniin also exhibited potent bioactivity at relatively non-toxic concentrations [61,69]. Preclinical in vivo studies further support a favorable safety profile, reporting radioprotective and antiviral effects in animal models [28,43]. However, these studies primarily focus on efficacy, and detailed toxicological parameters such as LD_50_, maximum tolerated dose, and long-term toxicity remain insufficiently characterized.

Clinical evidence is limited but suggests good short-term tolerability. A randomized single- and multiple-ascending-dose study of an ethanol extract (ES16001) in healthy volunteers reported only mild and transient adverse effects at doses up to 960 mg/day for 5 days, with no serious adverse events observed [68]. Notably, minor and reversible elevations in liver enzymes (ALT) were reported, indicating a need for further hepatic safety evaluation [68]. Despite these encouraging findings, significant gaps remain, including the absence of chronic toxicity, genotoxicity, reproductive toxicity, and comprehensive organ-specific safety assessments. Therefore, while current data suggest that E. sylvestris is relatively safe at tested doses, rigorous toxicological studies are required to establish its long-term safety and therapeutic index.

## 9. Gaps, Challenges, and Future Perspectives

Despite extensive pharmacological evidence, critical gaps remain in our understanding of *E. sylvestris*. Most studies are limited to in vitro or preliminary in vivo models, primarily focusing on antioxidant, anti-inflammatory, and antiviral effects, with insufficient exploration of molecular mechanisms. The specific molecular targets, receptor interactions, and downstream signaling cascades modulated by its principal bioactive constituents, such as geraniin (**1**), quercetin (**13**), and 1,2,3,4,6-penta-*O*-galloyl-*β*-D-glucose (**7**), remain largely uncharacterized. Moreover, while a few preclinical and early-phase clinical investigations, particularly for antiviral and radioprotective applications, have been reported, comprehensive human clinical trials regarding safety, efficacy, and dosage optimization are lacking.

Standardization and reproducibility of ESE pose another challenge. Variability in phytochemical content due to differences in geographic origin, extraction methods, and fermentation or roasting processes complicates consistency in pharmacological evaluation. Long-term toxicity, metabolism, pharmacokinetics, and potential herb–drug interactions are also underexplored, limiting translational development. Other pharmacological effects, such as anti-melanogenic, anti-atopic, antihypertensive, hepatoprotective, neuroprotective, and lipid-regulating activities, also require investigation. Future research should prioritize well-designed mechanistic studies that integrate systems biology, metabolomics, and molecular docking to elucidate structure–activity relationships. Rigorous in vivo models and clinical studies are needed to confirm therapeutic efficacy and safety. A multidisciplinary approach combining pharmacognosy, pharmacology, and biotechnology will be essential to fully realize the pharmaceutical and cosmeceutical potential of *E. sylvestris* and promote its rational development as a natural therapeutic resource.

## 10. Conclusions

*E. sylvestris* is a valuable medicinal plant with diverse pharmacological properties, including antioxidant, anti-inflammatory, antiviral, anticancer, and immunomodulatory effects, mainly attributed to polyphenolic constituents such as geraniin (**1**), quercetin (**13**), and penta-*O*-galloyl-*β*-D-glucose. It also shows promising cosmeceutical potential for skin protection and whitening. However, most studies remain confined to in vitro or preliminary in vivo models, with limited mechanistic and clinical evidence. Further molecular and clinical investigations are necessary to fully validate its therapeutic potential and support its development as a novel natural agent in medicine and cosmetics.

## Figures and Tables

**Table 2:** Anticancer effects of *E. sylvestris* extracts and compounds.

Compound/Extract	Source	Cancer Type/Cell Line	In Vitro/In Vivo	Treatment Concentration/IC_50_	Mechanism of Action	Key Effects	Reference
Hot water extract	Branches	MCF-7 (breast), HT-29 (colon)	In vitro	1–10 mg/mL	Cytotoxicity	Significant inhibition of proliferation (*p* < 0.05)	[40]
Hot water extract (fermented with *Pichia fermentans* or *Saccharomyces cerevisiae*)	Whole plant extract	HT-29 (colon), MCF-7 (breast), INT407 (intestinal)	In vitro	31–500 mg/mL	Anti-inflammatory, antioxidant	Reduced TNF-α and IL-6; cytotoxicity against HT-29 and MCF-7; improved INT407 viability	[41]
Cucurbitane-type triterpenoids, e.g., cucurbitacin D (**36**), 11-*O*-acetylmogroside I E1 (**32**)	Branches and leaves	HL-60 (leukemia), A549 (lung), SMMC-7721 (liver), MCF-7 (breast), SW480 (colon)	In vitro	IC_50_ values for: cucurbitacin D 0.06–1.20 µM IC_50_ values for 11-O-acetylmogroside I E1: 33–67 µM	Cytotoxicity via structural modifications	Strong cytotoxic effects; 11-*O*-acetyl group enhances activity	[36]
Methanolic extract	Leaves, branches	Detroit 551 (fibroblasts)	In vitro	100–500 μg/mL	Antioxidant, cytotoxicity	Suppressed H_2_O_2_ formation; cytotoxic effects; enhanced fibroblast proliferation	[42]
Extract rich in 1,2,3,4,6-penta-*O*-galloyl-β-D-glucose (**7**)	Leaves	Lymphocytes and intestinal crypt cells (mouse)	In vivo	10 mg/kg body weight (i.p.)	Anti-apoptotic, radioprotective	Reduced DNA damage and apoptosis; enhanced radioresistance	[43]

**Table 3:** Antioxidant effects of *E. sylvestris* extracts and compounds.

Compound/Extract	Source	Cell Line/Assay Type	In Vitro/In Vivo	Treatment Concentration/IC_50_	Mechanism of Action	Key Effects	Reference
1,2,3,4,6-penta-O-galloyl-β-D-glucose (**7**)	Leaves	Cell-free radical assays, H_2_O_2_-treated cells	In vitro	-	Radical scavenging, enzyme activity restoration	Quenched DPPH and ROS; restored SOD, CAT, GPx; inhibited lipid peroxidation; cytoprotective	[17]
Methanolic extract	Leaves, stem, and root	DPPH assay	In vitro	Leaves IC_50_ 1.86 μg/mL; stem 2.43 μg/mL; root 3.37 μg/mL	Radical scavenging	Highest phenolic content in leaves; potent antioxidant activity	[48]
Endophytic fungus *Pseudocercospora* sp. ESL 02	Leaves and stems	DPPH, reducing power, β-carotene bleaching	In vitro	IC_50_ DPPH: terreic acid 0.22 mmol/L, 6-methylsalicylic acid 3.87 mmol/L	Radical scavenging	Potent antioxidant compounds isolated from a fungus	[49]
Hot water extract (fermented with *Pichia fermentans* or *Saccharomyces cerevisiae*)	Whole plant extract	DPPH, ABTS assays	In vitro	31–500 mg/mL	Radical scavenging, metabolic modulation	Increased DPPH and ABTS activity; improved metabolomic profile	[41]
Hot water extract (fermented with *Lactobacillus kimchicus*/*L. plantarum*)	Whole plant extract	DPPH, ABTS assays	In vitro	-	Radical scavenging, polyphenol production	Enhanced antioxidant activity	[50]
Leaf extract–capped CuO nanoparticles	Leaves	DPPH, catalytic reduction assays	In vitro	-	Radical scavenging via nanoparticle-mediated electron transfer	Strong antioxidant efficiency	[51]
*E. Sylvestris*-mediated magnetite nanoparticles	Leaves	DPPH assay	In vitro	-	Radical scavenging	Antioxidant activity	[52]
Hot water extract	Branches	ABTS assay	In vitro	1–10 mg/mL	Radical scavenging	Higher antioxidant activity than *Magnolia officinalis*	[40]
Sub-critical water leaf extract	Leaves	DPPH, cosmetic-related assays	In vitro	-	Radical scavenging	Highest gallic acid content; strong antioxidant activity	[20]
Roasted aerial parts extract	Leaves and branches	DPPH, total phenol, total flavonoid	In vitro	-	Radical scavenging	Increased flavonoid and phenolic content; enhanced antioxidant activity	[31]

**Table 4:** Anti-inflammatory effects of *E. sylvestris* extracts and compounds.

Compound/Extract	Source	Cell Line/Animal Model	In Vitro/In Vivo	Treatment Concentration/Dose	Mechanism of Action	Key Effects	Reference
Ethanol extract	Leaves	RAW264.7 macrophages	In vitro	25–100 µg/mL	Downregulation of iNOS, COX-2, TNF-α, IL-1β, IL-6, IFN-γ; inhibition of NF-κB activation	Reduced NO and cytokine production	[56]
Ethanol extract		CIA mice	In vivo	25–100 mg/kg (alone or with SLZN 50 mg/kg)	Suppression of COX-2, iNOS, IL-1β, IL-6, TNF-α, MIP-1α, RANTES; inhibition of NF-κB	Decreased arthritis index, paw edema, serum cytokines	
Geraniin (**1**)		RAW264.7 macrophages	In vitro	≤40 µg/mL	Inhibition of iNOS, COX-2, and NF-κB pathway	Reduced NO and cytokine levels	
Elaeocarpusin (**2**)	Leaves	RBL-2H3, HMC-1, BMMCs, RPMCs	In vitro	0.01–10 µM	Inhibition of FcεRI phosphorylation (Fyn, Lyn, Syk, Akt); NF-κB blockade; reduced Ca^2+^ influx	Decreased histamine release and cytokine expression (TNF-α, IL-4)	[57]
Elaeocarpusin (**2**)		PCA model (mouse ear)	In vivo	10–100 ng/ear	Inhibition of mast cell degranulation and vascular permeability	Reduced ear edema and dye extravasation	
Elaeocarpusin (**2**)		OVA-induced ASA model (mouse)	In vivo	1–10 mg/kg (oral)	Suppression of serum histamine, IgE, and IL-4; inhibition of FcεRI and NF-κB signaling	Reduced hypothermia and allergic inflammation	
Hot water extract fermented with *Lactobacillus kimchicus*/*L. plantarum*	Whole plant	RAW264.7 cells	In vitro	-	NF-κB pathway modulation; polyphenols & organic acids	Reduced IL-6 and TNF-α; enhanced anti-inflammatory potential	[50]

**Table 6:** Anti-inflammatory effects of *E. sylvestris* extracts and compounds.

Compound/Extract	Source	Bacteria	In Vitro/In Vivo	Treatment Concentration/IC_50_	Mechanism of Action	Key Effects	Reference
Prodelphinidin oligomers	Bark	*S. aureus*, *Vibrio* spp.	In vitro	Minimum inhibitory concentration 98–389 µg/mL	Phenolic hydroxyl groups disrupt bacterial membranes	Strong antibacterial activity; 3,4,5-trihydroxyphenyl groups are crucial	[71]
Methanol extract	Leaves	*Helicobacter pylori* ATCC 43504	In vitro	1 mg/disk (≥20 mm zone)	Polyphenols disrupt bacterial membranes	Potent inhibition comparable to amoxicillin, tetracycline	[72]
Methanol extract, n-butanol fraction	Leaves, stems	*S. aureus*, *S. epidermidis*, *P. acnes*, *T. mentagrophytes*	In vitro	Zone of inhibition 23.3 mm	Phenolic/flavonoid compounds; membrane permeabilization	Higher activity than methylparaben and phenoxyethanol	[73]
Methanol extract, n-butanol fraction	Leaves	*S. aureus*	In vitro	Zone of inhibition 23.1–23.3 mm	Polar metabolites disrupt cell wall synthesis	High inhibition; stronger than synthetic antimicrobials	[74]
Ethanol and methanol extracts	Leaves, stems	*T. mentagrophytes*	In vitro	Clear zone <20.2 mm	Altered fungal membrane permeability	Moderate antifungal and antibacterial potential	[75]
ZnO nanoparticles	Leaves	*E. faecalis*, *P. aeruginosa*	In vitro	-	Reactive oxygen species (ROS) generation, metal oxide interaction	Strong antibacterial and photocatalytic activity	[76]
Magnetite nanoparticles	Leaves	*E. coli*, *S. aureus*	In vitro	-	ROS induction, Fe–O surface interactions	Inhibition of Gram-positive and Gram-negative bacteria	[52]
Methanol extract	Leaves, branches	*S. aureus*, *L. monocytogenes*, and lactic acid bacteria	In vitro	100 mg/mL	Polyphenolic antioxidants; cell wall disruption	Complete inhibition of S. aureus, reduced H_2_O_2_ formation	[77]
Methanol extract	Leaves, branches	*S. aureus*, *L. monocytogenes*	In vitro	100 mg/mL	Polyphenols/flavonoids	Complete inhibition; enhanced fibroblast proliferation	[42]

## Data Availability

No new data were created or analyzed in this study.

## Abbreviations

The following abbreviations are used in this manuscript.

*E. sylvestris*	*Elaeocarpus sylvestris*
ABTS	2,2′-azino-bis(3-ethylbenzothiazoline-6-sulfonic acid)
ALT	Alanine aminotransferase
ASA	Active systemic anaphylaxis
CIA	Collagen-induced arthritis
DPPH	2,2-diphenyl-1-picrylhydrazyl
ESE	*Elaeocarpus sylvestris* extract
EtOAc	Ethyl acetate
GPx	Glutathione peroxidase
HCMV	Human cytomegalovirus
HSV	Herpes simplex virus
IAV	Influenza A virus
IC_50_	Half maximal inhibitory concentration
IL	Interleukin
INT407	Human intestinal cell line
MCF-7	Michigan Cancer Foundation-7 (breast cancer cell line)
MIC	Minimum inhibitory concentration
NF-κB	Nuclear factor kappa B
PCA	Passive cutaneous anaphylaxis
ROS	Reactive oxygen species
SAD/MAD	Single ascending dose/Multiple ascending dose
SLZN	Sulfasalazine
SOD	Superoxide dismutase
SW480	Human colon cancer cell line
TNF-α	Tumor necrosis factor alpha
VZV	Varicella-zoster virus
